# Metabolomic profiling uncovers diagnostic biomarkers and dysregulated pathways in Parkinson’s disease

**DOI:** 10.3389/fneur.2025.1608031

**Published:** 2025-06-04

**Authors:** Hongfang Chen, Xing Cheng, Xiaoling Pan, Yu Yao, Lin Chen, Yaming Fu, Xinran Pan

**Affiliations:** ^1^Department of Neurology, The Affiliated Jinhua Hospital, Zhejiang University School of Medicine, Jinhua, Zhejiang, China; ^2^Key Laboratory of Neuropharmacology and Translational Medicine of Zhejiang Province, School of Pharmaceutical Sciences, The First Affiliated Hospital, Zhejiang Chinese Medical University, Hangzhou, Zhejiang, China; ^3^Central Laboratory, The Affiliated Jinhua Hospital, Zhejiang University School of Medicine, Jinhua, Zhejiang, China

**Keywords:** Parkinson’s disease, REM sleep behavior disorder, metabolomics, biomarkers, metabolic pathway

## Abstract

**Background:**

Parkinson’s disease (PD) is the second most common neurodegenerative disorder, and it has an unclear pathogenesis and lacks validated, specific biomarker-based diagnostic approaches, particularly in PD patients with rapid eye movement (REM) sleep behavior disorder (PD-RBD).

**Methods:**

Using untargeted liquid chromatography-mass spectrometry (LC–MS) metabolomics, serum profiles of 41 drug-naïve PD patients [including 19 PD-RBD and 22 PD without RBD (PD-nRBD) patients] and 20 healthy controls (HCs) were analyzed.

**Results:**

Comparative analyses revealed 144 dysregulated metabolites in PD patients versus HCs, with 7 metabolites—sodium deoxycholate, S-adenosylmethionine, L-tyrosine, 3-methyl-L-tyrosine, 4,5-dihydroorotic acid, (6Z)-octadecenoic acid, and allantoin—demonstrating high classification accuracy [area under the curve (AUC) > 0.93]. Compared with PD-nPBD patients, PD-RBD patients exhibited distinct metabolic profiles, characterized by 21 differentially expressed metabolites, including suberic acid, 3-methyl-L-tyrosine, and methyl (indol-3-yl) acetate (AUC > 0.86). Notably, 3-methyl-L-tyrosine displayed dual dynamics, reflecting dopaminergic depletion in PD and compensatory metabolic adaptations in PD-RBD. Pathway enrichment analysis implicated central carbon metabolism (CCM) disruption in PD and peroxisome proliferator-activated receptor (PPAR) signaling pathway inactivation in PD-RBD.

**Conclusion:**

These findings reveal potential serum-based biomarkers for PD and PD-RBD, highlight CCM and PPAR pathways as therapeutic targets, and underscore the role of metabolic dysregulation in PD pathophysiology.

## 1 Introduction

Parkinson’s disease (PD), now recognized as one of the leading causes of neurological disability (1), is pathologically characterized by aberrant *α*-synuclein aggregation and progressive degeneration of dopaminergic neurons in the substantia nigra (2). PD exhibits significant clinical heterogeneity, with phenotypes typically categorized according to the predominance of motor and non-motor symptom clusters (3). Although motor symptoms form the diagnostic cornerstone (4), non-motor manifestations—particularly rapid eye movement (REM) sleep behavior disorder (RBD), characterized by the loss of normal skeletal muscle atonia and vivid dream enactment during REM sleep (5, 6)—have emerged as critical markers of disease subtype stratification (1). Approximately 30–50% of PD patients have RBD (7), a phenotype that is associated with accelerated disease progression and a higher risk of cognitive impairment compared with PD patients without RBD (8). However, the diagnosis of PD remains clinically challenging due to its heavy reliance on subjective clinician-based evaluations and the absence of validated biomarkers for objectively diagnosing disease or identifying pathological changes.

Metabolomics, which focuses on small-molecule metabolites, has emerged as a promising strategy for molecular biomarker discovery, owing to its ability to detect pervasive metabolic dysregulations inherent in neurodegenerative pathologies (9, 10). In recent years, metabolomics has become an increasingly valuable tool in PD research, effectively connecting molecular mechanisms with dysregulated metabolic pathways and clinical manifestations that underlie the pathophysiology of PD. Multiple potential biomarkers for PD have been proposed, including 3-hydroxykynurenine (3-HK) (11), ornithine (12), 1-methylxanthine (13), hypoxanthine (14), caffeine and its metabolites (14), and lipid derivatives (15). However, there are no currently widely validated and used clinical biomarkers in peripheral blood. The clinical translation of these findings remains hindered by critical biological and methodological barriers, such as clinical heterogeneity, antiparkinsonian medication effects, analytical variability, and lack of robust multicenter validation. The pathogenic complexity of PD further complicates biomarker discovery. Accumulating data suggest that PD results from a dynamic interplay among senescence processes (16), inherited susceptibility, and environmental exposures (17), affecting numerous fundamental cellular processes, such as aberrant protein aggregation (18, 19), oxidative stress (20), neuroinflammation (21), and mitochondrial dysfunction (22, 23). Despite decades of research, the etiology of PD remains incompletely understood. Most existing studies have focused on comparing PD patients with healthy controls (HCs), with limited attention paid to the metabolic differences in PD patients with RBD.

In the present study, two comparative serum metabolomics analyses using untargeted liquid chromatography-mass spectrometry (LC–MS) metabolomics were conducted as follows: (i) drug-naïve PD patients versus HCs; and (ii) PD with RBD (PD-RBD) patients versus PD without RBD (PD-nRBD) patients. Our findings revealed potential diagnostic biomarkers and established precision phenotyping frameworks. The dual dynamics of 3-methyl-L-tyrosine highlighted phenotype-specific metabolic adaptations. Moreover, the present results revealed central carbon metabolism (CCM) disruption in PD and PPAR signaling inactivation in PD-RBD, linking metabolic dysfunction to neurodegeneration and highlighting CCM and PPAR signaling pathways as therapeutic targets. Future work requires multicenter validation and multiomics integration to translate these insights into clinical applications.

## 2 Materials and methods

### 2.1 Participants

Participants were recruited from the Outpatient Department of the Affiliated Jinhua Hospital of Zhejiang University School of Medicine, including 61 individuals (41 patients with PD and 20 HCs). All patients with PD were newly diagnosed according to the Movement Disorder Society (MDS) Clinical Diagnostic Criteria for PD (MDS-PD Criteria) and were drug-naïve, having not initiated any antiparkinsonian medications prior to enrollment. The exclusion of secondary parkinsonian syndromes was confirmed by normal findings on 3.0-Tesla brain magnetic resonance imaging (3.0 T MRI), which revealed intact nigrostriatal pathways (without evidence of vascular lesions, midbrain atrophy, or iron deposition in globus pallidus). The clinical baseline of PD patients was assessed by two movement disorder specialists (H.F.C. and X.L.P.), using the Unified Parkinson’s Disease Rating Scale (UPDRS III), Hoehn and Yahr (H-Y) staging, the RBD screening questionnaire (RBDSQ), and the Mini-Mental State Examination (MMSE). In addition, PD patients were stratified into the following two subgroups based on the RBDSQ scores, namely, PD-RBD (RBDSQ score ≥ 6) and PD-nRBD (RBDSQ score < 6), using the validated cutoff of 6 points for clinical relevance (24, 25). Age- and sex-matched HCs underwent standardized neurological evaluations to confirm the absence of neurological disorders. All enrolled participants (both PD patients and healthy controls) were free of infections, hepatic dysfunction, renal dysfunction, hypertension, diabetes mellitus, neoplasms, and autoimmune diseases. All participants were free of any medications (including over-the-counter drugs, vitamins, nutraceuticals, or herbal supplements) for at least eight weeks prior to blood collection. Each participant signed a written informed consent before enrollment, and this study received approval from the Ethics Committee of the Affiliated Jinhua Hospital, Zhejiang University School of Medicine [Approval no. (Research) 2022-Ethical Review-221, date: September 7, 2022]. This research was conducted following the ethical principles of the Declaration of Helsinki.

### 2.2 Serum sample collection and processing

Venous blood samples were collected from all participants in the morning following an overnight fast of at least 12 h (8: 00 PM to 8:00 AM) (26). During the fasting period, participants were allowed to consume small amounts of pure water until 10:00 PM. The serum was separated within 60 min after collection by centrifugation at 2000 × g for 10 min and subsequently stored at −80°C until further analysis.

Samples were processed for metabolite extraction according to previously reported methods (27). In brief, serum samples were thawed at 4°C and then vortexed for 1 min to ensure complete homogenization. Then, 50 μL of serum was mixed with 400 μL of methanol in a 2 mL centrifuge tube. After vortexing for 1 min and centrifugation at 12,000 × g for 10 min at 4°C, the supernatant was transferred to a new 2 mL centrifuge tube. The sample was then concentrated and dried. Finally, 150 μL of 2-chloro-l-phenylalanine (4 ppm) solution prepared with 80% methanol in water was added to redissolve dried extracts. The solution was then filtered through a 0.22 μm membrane and transferred to a detection bottle for LC–MS analysis. Quality control (QC) samples were prepared by mixing 10 μL of each extracted serum sample to monitor the LC–MS instrument stability.

### 2.3 LC–MS analysis

LC–MS analysis was performed on a Vanquish UHPLC System (Thermo Fisher Scientific, USA).

Chromatographic separation was performed using an ACQUITY UPLC^®^ HSS T3 column (2.1 × 100 mm, 1.8 μm; Waters, Milford, MA, USA) maintained at 40°C. The flow rate was 0.3 mL/min, and the injection volume was 2 μL. For LC-ESI(+)-MS (positive ion mode) analysis, the mobile phases consisted of 0.1% (v/v) formic acid in water (A1) and 0.1% (v/v) formic acid in acetonitrile (B1). For LC-ESI(−)-MS (negative ion mode) analysis, the mobile phases were 5 mM ammonium formate in water (A2) and acetonitrile (B2). Both analyses were conducted under the same elution gradient (28) as follows: 0–1 min, 8% B; 1–8 min, 8–98% B; 8–10 min, 98% B; 10–10.1 min, 98–8% B; and 10.1–12 min, 8% B.

Mass spectrometric detection of metabolites utilized an Orbitrap Exploris 120 instrument (Thermo Fisher Scientific, USA) equipped with an ESI ion source. Data acquisition employed full-scan MS1 (m/z 100–1,000) at 60,000 FWHM, followed by data-dependent MS/MS (ddMS2) scans at 15,000 FWHM. Source parameters included sheath gas pressure (40 arb), auxiliary gas flow (10 arb), spray voltage (+3.5 kV for ESI[+] and-2.5 kV for ESI[−]), capillary temperature (325°C), number of data-dependent scans per cycle (4), normalized collision energy (30%), and dynamic exclusion time (automatic) (29).

### 2.4 Data processing and metabolite identification

Prior to analysis, raw metabolite intensities underwent total peak area normalization followed by log2 transformation to improve normality. The raw LC–MS data were firstly converted to mzXML format by MSConvert in ProteoWizard software package (30) (v3.0.8789) and processed using XCMS (version 3.12.0) in R for feature detection, retention time correction, and alignment (31), yielding a quantitative list of metabolites. Metabolites exhibiting a relative standard deviation (RSD) > 30% in QC samples were excluded, while the remaining metabolites were retained for subsequent analysis (29).

Metabolites were identified using MS1 and MS/MS spectra against the following databases: the Human Metabolome Database (HMDB) (32), Kyoto Encyclopedia of Genes and Genomes (KEGG) (33), LipidMaps (34), MassBank (35), mzCloud (36), and the metabolite database built by Biomedical Tech Co., Ltd. (Suzhou, China). Primary identification was achieved by matching precursor ion m/z (mass error tolerance < 30 ppm) and adduct information to derive molecular formulas. The quantitative metabolites with MS/MS spectra were compared and matched to the fragment ion information of each MS/MS spectrum in these databases to achieve the secondary identification of these metabolites.

### 2.5 Statistical and pathway analyses

All statistical analyses were performed using R statistical software (version 4.3.1). The orthogonal partial least squares discriminant analysis (OPLS-DA) model was employed to evaluate group separation and clustering (37–39). The R^2^ (model explainability) and Q^2^ (model predictability) were calculated to assess the stability and reliability of the model by 7-fold cross-validation (40). In 7-fold cross-validation, the dataset was randomly partitioned into seven equally sized subsets, with each subset iteratively serving as the validation set while the remaining six subsets were used for model training (41). A variable importance in projection (VIP) score threshold > 1 was used to extract the significant contributor metabolites to group separation in the OPLS-DA model (42).

Differential metabolites between groups were identified using Student’s independent t-tests, with statistical significance defined as *p* < 0.05. Multiple comparison adjustments were implemented through the Benjamini-Hochberg procedure with a false discovery rate (FDR) < 0.05. Fold change (FC) values were calculated as the median intensity ratio between groups (PD vs. HC and PD-RBD vs. PD-nRBD). Volcano plots were used to visualize metabolite significance [−log10 (*p*-value)] and magnitude of FC [log2(FC)]. Hierarchical biclustering analysis was applied to both samples and metabolites, generating clustered heatmaps. Receiver operating characteristic (ROC) curves were constructed, and the area under the curve (AUC) was computed to evaluate biomarker diagnostic performance. AUC values were interpreted as follows: 0.9–1.0 (excellent), 0.8–0.9 (good), 0.7–0.8 (fair), 0.6–0.7 (poor), and <0.6 (fail) (43). Age, UPDRS part III, H-Y stage, RBDSQ, and MMSE were compared between groups using Student’s independent t-tests. Sex composition was analyzed via a chi-squared test. All quantitative data are presented as the means ± standard deviations (SDs) unless specified otherwise.

Significantly altered metabolites were analyzed for pathway enrichment using MetaboAnalyst (44),^1^ followed by mapping onto KEGG pathways to elucidate higher-level systemic functional implications. Visualizations of metabolite-pathway associations were generated through the KEGG Mapper tool.

## 3 Results

### 3.1 Comparison of demographic and clinical variables of participants

The demographic characteristics of the PD and HC groups are presented in Table 1. There were no significant differences in age distribution or sex composition. The demographic and clinical features of PD-RBD and PD-nRBD patients are detailed in Table 2. Age, sex composition, UPDRS part III, H-Y stage, and MMSE scores did not show significant differences between the PD-RBD and PD-nRBD groups, but there was a significant difference in the RBDSQ scores between the groups.

**Table 1:** Demographic data for recruited patients with PD and HCs.

Demographic characteristics	PD (*n* = 41)	HC (*n* = 20)	*p*-value^a^
Age (years)	63.97 ± 9.09	61.70 ± 13.14	0.43
Sex (F/M)	21/20	11/9	0.78

PD, Parkinson’s disease; HC, healthy control; F/M, female and male; Values are presented as the means ± SDs. ^a^*p*-value using a Student’s independent *t*-test for age and a chi-squared test for sex composition.

**Table 2:** Demographic and clinical data of PD-RBD and PD-nRBD.

Demographic and clinical characteristics	PD-RBD (*n* = 19)	PD-nRBD (*n* = 22)	*p*-value^a^
Age (years)	64.42 ± 8.80	63.59 ± 9.53	0.78
Sex (F/M)	9/10	12/10	0.65
UPDRS part III	26.68 ± 10.61	26.82 ± 14.79	0.97
H-Y stage	1.89 ± 0.72	1.98 ± 0.61	0.69
RBDSQ	8.05 ± 2.17	2.00 ± 0.87	<0.001
MMSE	25.00 ± 3.73	23.77 ± 5.73	0.43

PD-RBD, PD with RBD patients; PD-nRBD, PD without RBD patients; F/M, female and male; UPDRS part III, Unified Parkinson’s Disease Rating Scale III; H-Y stage, Hoehn and Yahr stage; RBDSQ, RBD screening questionnaire; MMSE, Mini-Mental State Examination; Values are presented as the means ± SDs. ^a^*p*-value using Student’s independent *t*-tests for age, UPDRS part III, H-Y stage, RBDSQ, MMSE, and a chi-squared test for sex composition.

### 3.2 Metabolic signatures of drug-naïve PD patients compared to HCs

**Figure 1:**
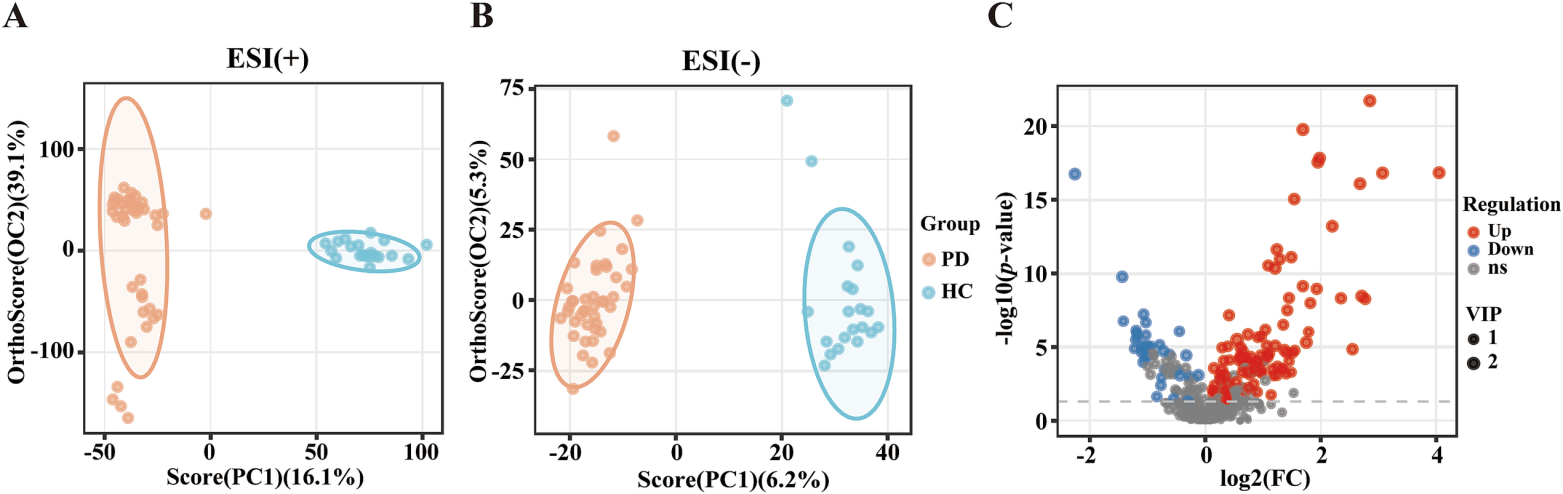
Altered metabolic profiles between patients with PD and HCs. **(A)** OPLS-DA score plot. ESI (+): positive ion mode, R^2^X = 0.599, R^2^Y = 0.967, Q^2^ = 0.927. **(B)** OPLS-DA score plot. ESI (−): negative ion mode, R^2^X = 0.115, R^2^Y = 0.972, Q^2^ = 0.827. **(C)** Volcano plot of metabolites in the PD group versus the HC group. Red: upregulated metabolite; Blue: downregulated metabolite; Grey: metabolite not meeting the significance thresholds.

To investigate the differential metabolites in PD patients, the serum metabolites were introduced to OPLS-DA models. The metabolites of PD patients were clearly separated from HCs on the OPLS-DA score plots [ESI(+): R^2^X = 0.599, R^2^Y = 0.967, Q^2^ = 0.927; ESI(−): R^2^X = 0.115, R^2^Y = 0.972, Q^2^ = 0.827] (Figures 1A,B). Among the 425 metabolites, 144 metabolites exhibited significant distinction between PD and HC groups (VIP scores > 1), with 107 upregulated metabolites (represented by red dots) and 37 downregulated metabolites (represented by blue dots) in PD patients relative to HCs. The volcano plot provided a graphical representation of the significance and magnitude of changes in metabolite levels, highlighting the most prominent alterations in the PD group (Figure 1C). Univariate analysis with FDR correction revealed 132 different metabolites between the PD and HC groups (FDR < 0.05). These metabolites included mainly lipids, amino acids and their derivatives, organic acids and their derivatives, nucleotides and their derivatives, carbohydrates and their derivatives, terpenoids, sterols, vitamins, cofactors, alkaloids compounds, nitrogen compounds, and phenolic compounds (Supplementary Table S1).

### 3.3 Identification of potential metabolic biomarkers for PD

**Figure 2:**
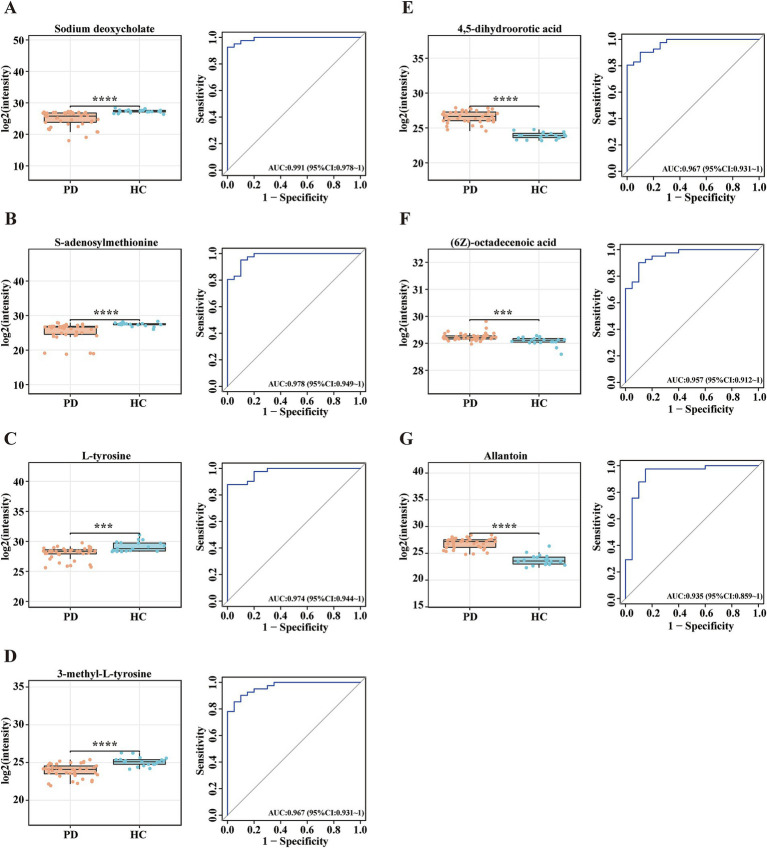
Potential metabolite biomarkers for PD diagnosis. (A-G) Box plots and ROC curves for the serum levels of **(A)** sodium deoxycholate, **(B)** S-adenosylmethionine, **(C)** L-tyrosine, **(D)** 3-methyl-L-tyrosine, **(E)** 4,5-dihydroorotic acid, **(F)** (6Z)-octadecenoic acid, and **(G)** allantoin for the diagnosis of PD. Data are expressed as the means ± SDs. ***p ≤ 0.001 and ****p ≤ 0.0001.

To evaluate the diagnostic potential of serum metabolites in PD, ROC curve analysis was applied to metabolomic profiles derived from PD patients and HCs. The AUC values of ROC curves were used to assess the diagnostic potential of the identified metabolites. Among the 132 selected metabolites, sodium deoxycholate had the greatest ability (AUC = 0.991, Figure 2A) to differentiate PD patients from HCs, followed by S-adenosylmethionine (AUC = 0.978, Figure 2B), L-tyrosine (AUC = 0.974, Figure 2C), 3-methyl-L-tyrosine (AUC = 0.967, Figure 2D), 4,5-dihydroorotic acid (AUC = 0.967, Figure 2E), (6Z)-octadecenoic acid (AUC = 0.957, Figure 2F), and allantoin (AUC = 0.935, Figure 2G). The *p*-values for all selected metabolites were statistically significant (*p* < 0.001). Compared with the HC group, the concentrations of sodium deoxycholate, S-adenosylmethionine, L-tyrosine, and 3-methyl-L-tyrosine were lower in the PD group, while the concentrations of 4,5-dihydroorotic acid, (6Z)-octadecenoic acid, and allantoin were higher in the PD group (Table 3). This analysis identified seven candidate metabolites with significant discriminatory power, highlighting their potential as biomarkers for PD diagnosis.

**Table 3:** Identification of biomarkers between patients with PD and HCs.

Biomarkers	Molecular formula	Measured m/z	RT (s)	ppm	VIP	log2(FC)	*p*-value^a^	AUC	Trend	ESI mode
Sodium deoxycholate	C₂₄H₃₉O₄Na	415.2105	465.3	0.293	1.259	−1.42	<0.0001	0.991	↓	ESI+
S-adenosylmethionine	C₁₅H₂₂N₆O₅S	398.2395	459.6	3.317	1.079	−1.1	<0.0001	0.978	↓	ESI+
L-tyrosine	C₉H₁₁NO₃	182.0807	74.1	2.614	1.254	−1.08	0.0001	0.974	↓	ESI+
3-methyl-L-tyrosine	C₁₀H₁₃NO₃	195.1016	485.5	0.025	1.455	−1.07	<0.0001	0.967	↓	ESI+
4,5-dihydroorotic acid	C₅H₆N₂O₄	158.9611	189.9	0.706	2.157	2.85	<0.0001	0.967	↑	ESI+
(6Z)-octadecenoic acid	C₁₈H₃₄O₂	281.2479	663.8	0.016	1.684	0.15	0.0003	0.957	↑	ESI-
Allantoin	C₄H₆N₄O₃	158.9607	88.2	18.197	2.159	3.07	<0.0001	0.935	↑	ESI+

PD, Parkinson’s disease; HC, healthy control; RT, retention time; ppm, part per million; VIP, variable important in projection; FC, fold change; AUC, area under the curve; ↑: up-regulation; ↓: down-regulation; ESI+/−: Positive ion mode/Negative ion mode; ^a^*p*-value obtained Student’s independent *t*-tests.

### 3.4 Metabolomic analysis reveals distinct metabolic profiles in PD-RBD compared to PD-nRBD

**Figure 3:**
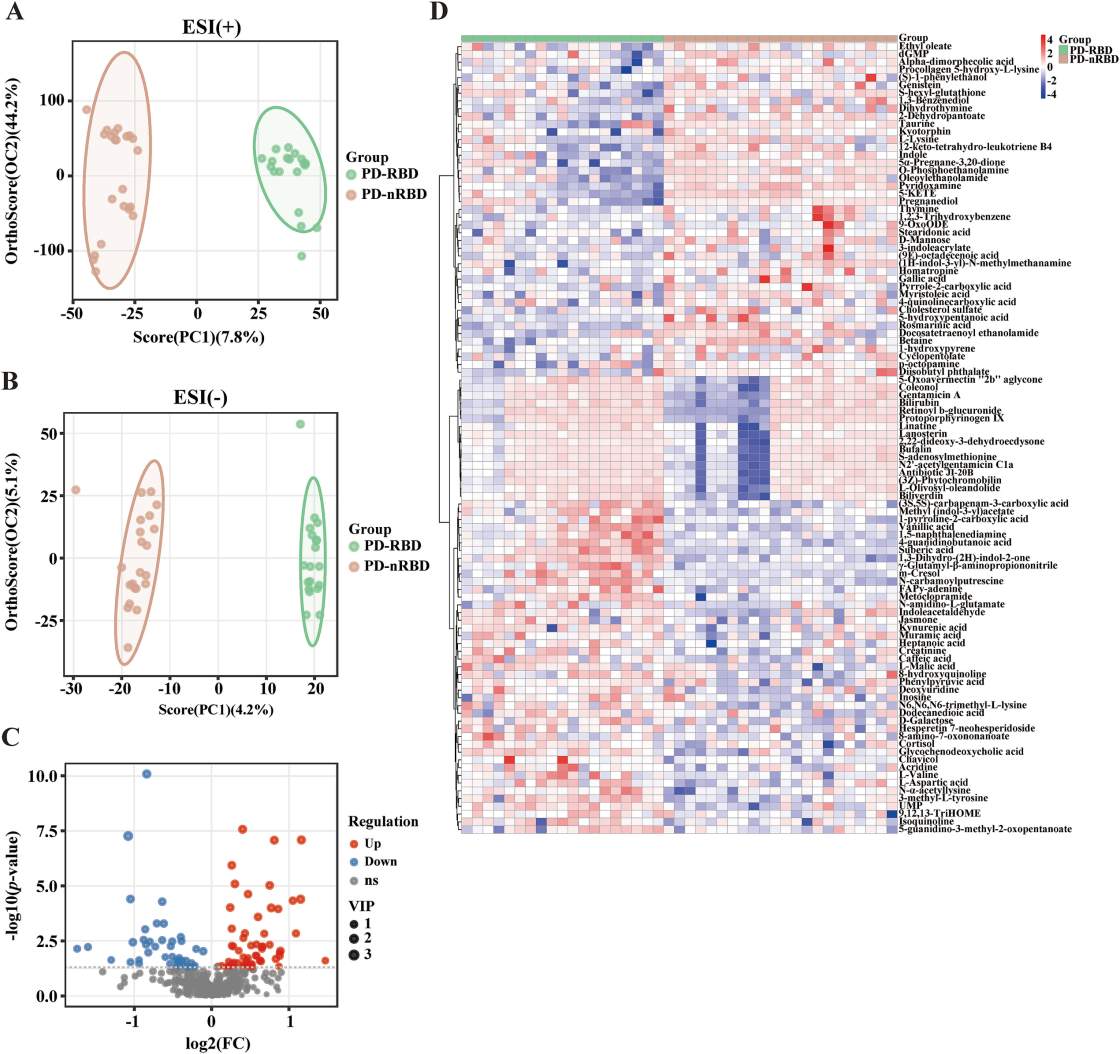
Altered serum metabolites of PD-RBD compared to PD-nRBD. **(A)** OPLS-DA score plots. ESI (+): positive ion mode, R^2^X = 0.564, R^2^Y = 0.974, Q^2^ = 0.758. **(B)** OPLS-DA score plot. ESI (−): negative ion mode, R^2^X = 0.0938, R^2^Y = 0.981, Q^2^ = 0.536. **(C)** Volcano plot of upregulated (red) and downregulated (blue) metabolites in the PD-RBD group versus the PD-nRBD group. **(D)** Heatmap of the 102 differential metabolites in the PD-RBD group versus the PD-nRBD group. Red indicates an increased level, and blue indicates a decreased level.

**Figure 4:**
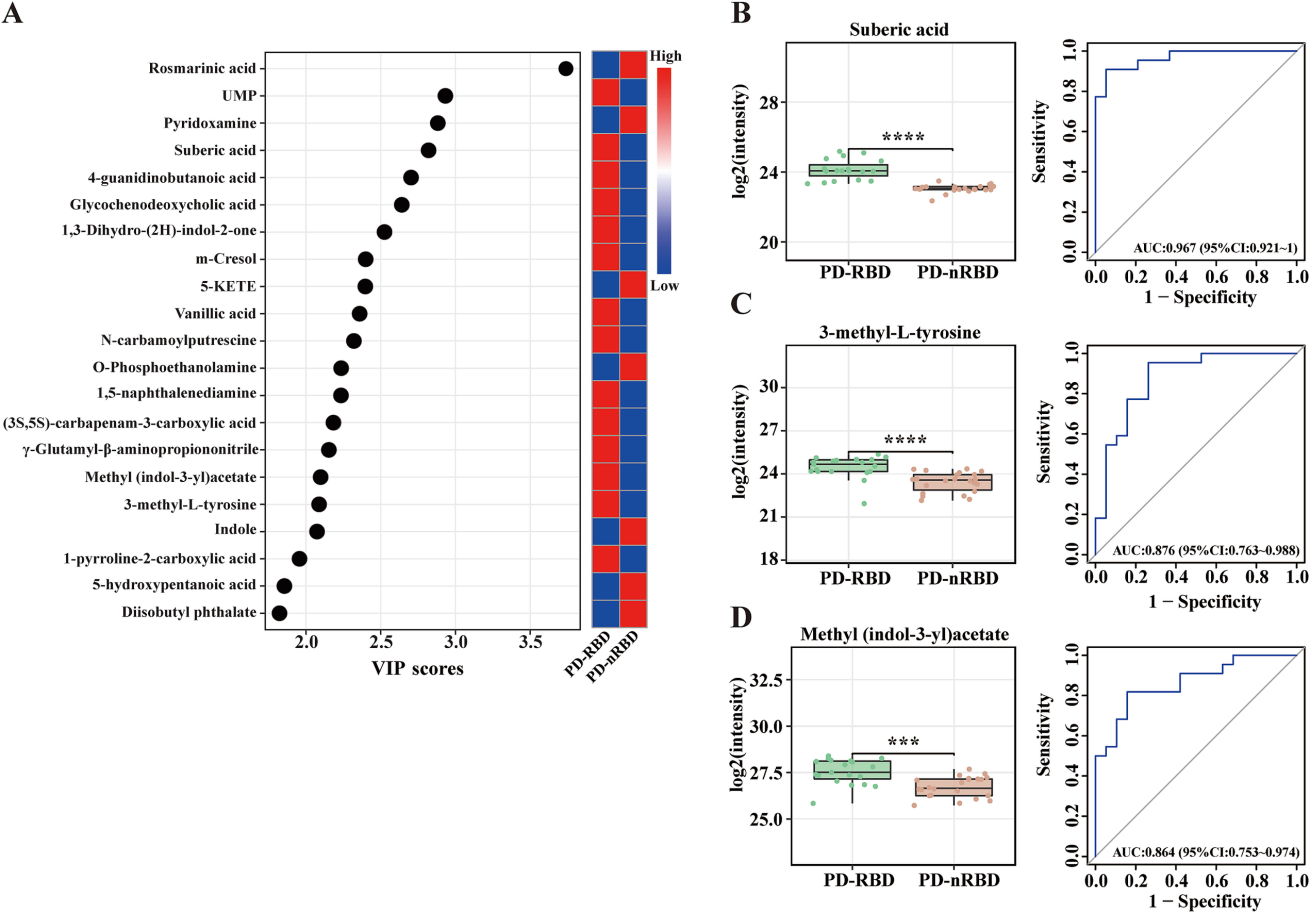
Significantly altered metabolite clusters and potential metabolite biomarkers for PD-RBD. **(A)** Twenty-one metabolites had VIP > 1 (also with FDR < 0.05), indicating their contribution to the classification in the OPLS-DA score plot. **(B–D)** Box plots and ROC curves for the serum levels of **(B)** suberic acid, **(C)** 3-methyl-L-tyrosine, and **(D)** methyl (indol-3-yl)acetate for the diagnosis of PD patients with RBD. Data are expressed as the means ± SD. ***p ≤ 0.001 and ****p ≤ 0.0001.

The OPLS-DA score plots for all serum metabolites demonstrated clear separation between the PD-RBD and PD-nRBD groups. Additionally, the OPLS-DA score plots exhibited high separative and predictive validity, with robust R^2^Y and Q^2^ values in the positive ion mode [ESI(+): R^2^Y = 0.974, Q^2^ = 0.758] and negative ion mode [ESI(−): R^2^Y = 0.981, Q^2^ = 0.536], respectively (Figures 3A,B). The volcano plot revealed distinct regulatory patterns among these metabolites, with 59 upregulated metabolites (represented by red dots) and 43 downregulated metabolites (represented by blue dots) validated through the OPLS-DA score plots (Figure 3C). Furthermore, the heatmap shown in Figure 3D illustrates the differential expression patterns of the 102 metabolites (VIP scores > 1.0, *p* < 0.05) in the PD-RBD group compared with the PD-nRBD group. Among these, 21 metabolites (FDR < 0.05) displayed significant differential abundance between the PD-RBD and PD-nRBD groups. These differentially expressed metabolites were predominantly categorized as seven secondary metabolites, five amino acid derivatives, four lipids, two organic acids, one cofactor, one nucleotide, and one aromatic amine, as shown in the VIP score analysis (Figure 4A).

Next, we conducted ROC curve analyses for the 21 selected metabolites to further evaluate their potential as diagnostic biomarkers for patients with PD-RBD. Notably, suberic acid exhibited the highest diagnostic accuracy (AUC = 0.967, Figure 4B), followed by 3-methyl-L-tyrosine (AUC = 0.876, Figure 4C) and methyl (indol-3-yl)acetate (AUC = 0.864, Figure 4D). The concentrations of these metabolites were higher in the PD-RBD group than in the PD-nRBD group (Table 4). These findings highlighted the potential of these three metabolites as candidate biomarkers for diagnosing patients with PD-RBD and provided insights into the metabolic pathways that may be involved in the pathogenesis of RBD within the context of PD.

**Table 4:** Identification of biomarkers between PD-RBD and PD-nRBD.

Biomarkers	Molecular formula	Measured m/z	RT (s)	ppm	VIP	log2(FC)	*p*-value^a^	AUC	Trend	ESI mode
Suberic acid	C₈H₁₄O₄	157.0834	135.3	0.162	2.820	1.16	<0.0001	0.967	↑	ESI+
3-methyl-L-tyrosine	C₁₀H₁₃NO₃	195.1016	485.5	0.025	2.088	1.05	<0.0001	0.876	↑	ESI+
Methyl (indol-3-yl)acetate	C₁₁H₁₁NO₂	172.0715	675	0.384	2.098	0.86	0.0001	0.864	↑	ESI+

PD-RBD, PD with RBD patients; PD-nRBD, PD without RBD patients; RT, retention time; ppm, part per million; VIP, variable important in projection; FC, fold change; AUC, area under the curve; ↑: up-regulation; ↓: down-regulation; ESI+/−: Positive ion mode/Negative ion mode; ^a^*p*-value obtained Student’s independent *t*-tests.

### 3.5 Metabolic pathway enrichment analysis

**Figure 5:**
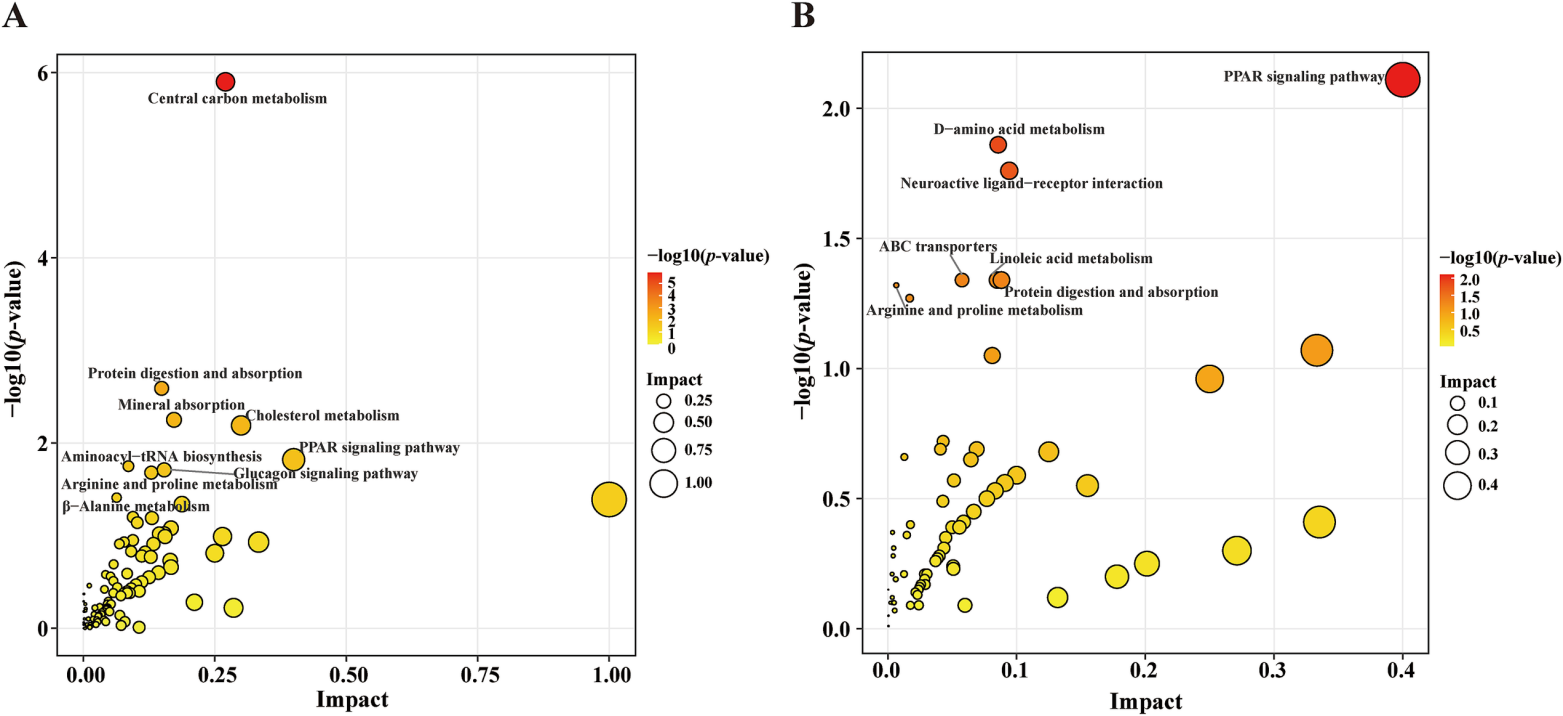
Scatter plot showing the KEGG pathway enrichment analysis results. **(A)** Pathway analysis of the significantly altered metabolites in the PD group versus the HC group. The red-to-yellow gradient signifies ascending *p*-values, and the dot size is scaled to show the magnitude per pathway. **(B)** Pathway analysis of the significantly altered metabolites in the PD-RBD group versus the PD-nRBD group.

Compared with the HC group, KEGG pathway analysis of the differentially abundant metabolites identified significant enrichment (*p* < 0.05) of the following nine metabolic pathways in the PD group: CCM; protein digestion and absorption; mineral absorption; cholesterol metabolism; PPAR signaling pathway; aminoacyl-tRNA biosynthesis; glucagon signaling pathway; arginine and proline metabolism; and beta-alanine metabolism (Figure 5A). Compared with the PD-nRBD group, KEGG pathway analysis of the altered metabolites revealed significant enrichment (*p* < 0.05) of the following seven pathways in the PD-RBD group: PPAR signaling pathway; D-amino acid metabolism; neuroactive ligand-receptor interaction; protein digestion and absorption; linoleic acid metabolism; ABC transporters; and arginine and proline metabolism (Figure 5B).

## 4 Discussion

The present study utilized untargeted LC–MS analysis to investigate serum metabolic profiles in drug-naïve PD patients compared to HCs. The metabolites that significantly decreased in the PD group included L-tyrosine and S-adenosylmethionine. L-tyrosine, a primary precursor of dopamine, plays a crucial role in dopamine (DA) synthesis (45), and the depletion of L-tyrosine indirectly reflects DA deficiency in the nigrostriatal pathway. A previous study reported similar tyrosine levels between levodopa-treated PD patients and healthy controls (46), which contrasts with our observation of reduced L-tyrosine in a drug-naïve PD cohort. This discrepancy may reflect the modulatory effects of levodopa therapy on tyrosine metabolism. L-tyrosine faces therapeutic challenges due to the blood–brain barrier, while its downstream metabolite, L-Dopa, is used to supplement DA substrates in the brain (47). The limitations of DA replacement therapy, such as diminishing efficacy and drug-induced motor complications (48), highlight the need to reconsider its upstream metabolite, L-tyrosine. Specifically, targeting L-tyrosine metabolism may offer novel opportunities to enhance DA synthesis through the upregulation of tyrosine hydroxylase (TH) activity using genetic engineering techniques. Similarly, S-adenosylmethionine serves as a principal methyl donor in epigenetic regulation (49), glutathione synthesis (50), and neurotransmitter synthesis (including DA metabolism) (51). Consistent with previous findings demonstrating significantly reduced S-adenosylmethionine levels in PD patients compared to control subjects (51–53), the observed S-adenosylmethionine depletion in our study may indicate a pathology of impaired methylation capacity, increased oxidative stress, and mitochondrial dysfunction, collectively contributing to *α*-synuclein aggregation and progressive neurodegeneration. Additionally, S-adenosylmethionine restricts the expression of A_2A_ receptors, which are upregulated in PD patients, thereby indirectly enhancing DA signaling (54–57). This suggests that S-adenosylmethionine replenishment strategies may have a specific targeted effect on A_2A_ receptors in the brain and could synergize with existing dopaminergic therapies. Moreover, we observed significantly elevated serum allantoin levels in PD patients compared with HCs, which is consistent with previous metabolomic findings (8), indicating increased oxidative stress in synucleinopathy. The levels of sodium deoxycholate, 4,5-dihydroorotic acid, and (6Z)-octadecenoic acid were also significantly altered in the PD group, which may reflect underlying pathological processes in PD, such as gut dysbiosis (58), energy metabolism dysfunction (59), and inflammation (60). These findings demonstrate the complexity of metabolic disturbances in PD, suggesting that these metabolites could serve as both diagnostic biomarkers and potential therapeutic targets.

KEGG pathway analysis revealed that CCM was the most significantly altered pathway in PD pathology, with the highest number of PD-associated metabolic changes localized to this category (e.g., L-malic acid, citric acid, and isocitrate), consistent with findings from previous studies (61, 62). CCM, traditionally encompassing the glycolytic pathway [Embden-Meyerhof-Parnas (EMP) pathway], the pentose phosphate pathway (PPP), and the tricarboxylic acid (TCA) cycle, serves as the core of energy production and also as a hub connecting lipid and amino acid metabolism (63). Dysregulation of this pathway underscores insufficient energy and mitochondrial dysfunction in PD (4). For example, reduced levels of L-malic acid impair the TCA cycle, resulting in decreased nicotinamide adenine dinucleotide (NADH, reduced form) production and adenosine triphosphate (ATP) synthesis, ultimately inhibiting oxidative phosphorylation in mitochondria. Thus, the pathway enrichment analysis highlighted the potential role of CCM in the neurodegenerative process of PD and provided a basis for further investigation into the underlying mechanisms. To bridge these findings to clinical applications, a heterogeneous information network (HIN) learning model integrating multi-omics data (e.g., metabolomics, proteomics, and mitochondrial genomics) could map PD-specific CCM bottlenecks (e.g., malate dehydrogenase dysfunction) to prioritize therapeutic targets (64). For instance, 3D molecular pocket-based generation techniques could design small molecules to allosterically activate TCA cycle enzymes, compensating for L-malic acid depletion and restoring NADH/ATP production (65).

Further analysis of differential serum metabolites between the PD-RBD and PD-nRBD groups revealed that only 3 out of 102 differentially expressed metabolites associated with PD-RBD demonstrated potential as biomarkers (AUC > 0.86). Methyl (indol-3-yl)acetate, a derivative of indole-3-acetic acid (66), is associated with the tryptophan metabolic pathway (67)—encompassing serotonin and melatonin synthesis—which is critically implicated in sleep regulation and mood disorders (68–70). The elevation of methyl (indol-3-yl)acetate in PD-RBD patients suggested alterations in gut microbiota composition or function, leading to disturbances in the tryptophan metabolic pathway and potentially contributing to sleep–wake cycle dysregulation. This dysregulation may reflect a gut-brain axis dysfunction, as altered microbial tryptophan metabolism modulates systemic levels of neuroactive metabolites (71). In PD, the propagation of *α*-synuclein pathology from the gut to the brain (Braak’s hypothesis) (72) may be exacerbated by gut dysbiosis (73, 74). Suberic acid, an aliphatic dicarboxylic acid, was significantly elevated in PD-RBD patients, indicating impaired fatty acid *β*-oxidation and exacerbation of neuronal energy deficits. Notably, increased suberic acid levels have also been observed in the urine metabolites of PD patients, further supporting the role of mitochondrial energy metabolism dysregulation in PD-related pathology (75, 76). This impaired fatty acid β-oxidation could lead to ATP depletion, thereby impairing synaptic function and exacerbating neurodegeneration in vulnerable regions such as the substantia nigra—a key site affected in PD (77). Moreover, suberic acid accumulation could promote reactive oxygen species (ROS) overproduction, exacerbating oxidative stress that facilitates α-synuclein misfolding and aggregation (78, 79). This mechanism supports the established pathological association between mitochondrial ROS generation and α-synucleinopathy—a pathological hallmark of PD—in synucleinopathies (80–82). In contrast, 3-methyl-L-tyrosine exhibited a dual pattern, with decreased serum levels in PD patients compared with HCs and increased levels in PD-RBD patients compared with PD-nRBD patients. As a methylated derivative of L-tyrosine (83), the significant reduction in 3-methyl-L-tyrosine levels in the PD group aligns with the observed decline in L-tyrosine levels in our study, thereby providing another perspective on dopaminergic depletion. Notably, previous studies have demonstrated that PD patients receiving levodopa therapy exhibit significantly elevated serum levels of 3-methyl-L-tyrosine compared to healthy controls (2, 46), whereas our drug-naïve cohort exhibited the opposite trend. This contrast suggests that L-Dopa may modulate tyrosine metabolism through alternative pathways or altered enzymatic activity during dopaminergic replacement therapy. In PD-RBD patients, TH activity is more severely reduced compared with PD-nRBD patients (84). This pronounced TH deficiency leads to impaired conversion of tyrosine to L-Dopa, thereby disrupting DA biosynthesis. Consequently, such metabolic blockage may redirect tyrosine flux toward alternative pathways, resulting in the accumulation of tyrosine-derived intermediates—such as 3-methyl-L-tyrosine—in the systemic circulation. This duality underscores the dynamic interplay between neurodegeneration and metabolic adaptation across PD progress.

Pathway enrichment analysis identified the PPAR signaling pathway as a key dysregulated pathway in PD-RBD, with a tendency towards inactivation. PPARs are nuclear receptors that modulate lipid metabolism, inflammation, cellular differentiation, and mitochondrial biogenesis (85, 86). The present findings of altered metabolites involved in lipid metabolism, such as alpha-dimorphecolic acid, align with the involvement of PPAR signaling in these conditions. Dysregulation of the PPAR signaling pathway has been implicated in the pathogenesis of PD (86, 87), as it plays a crucial role in energy metabolism (88), antioxidant stress response (89), and circadian metabolic homeostasis (90). For example, PPARα agonists demonstrate neuroprotective effects in 1-methyl-4-phenyl-1,2,3,6-tetrahydropyridine (MPTP)-induced PD mice by attenuating oxidative stress (89). Additionally, the deletion of PPARγ has been shown to disrupt diurnal rhythms in mice (91)—a dysfunction particularly relevant to the progression of RBD symptoms in PD. These findings collectively suggest that targeting the PPAR signaling pathway may alleviate metabolic disturbances in PD-RBD. Coupled with large language models (LLMs) trained on biomedical literature and clinical trial databases, researchers could rapidly screen FDA-approved drugs for repurposing candidates (e.g., anti-diabetic agents targeting PPARγ) that mitigate both motor and non-motor symptoms in PD-RBD (92). To optimize therapeutic efficacy, geometric deep learning (GDL) could predict drug–drug associations (DDAs) within a PPAR-centered heterogeneous network (93). By analyzing the geometric relationships between PPAR agonists, mitochondrial enhancers, and circadian modulators, GDL models may identify synergistic combinations (e.g., pioglitazone with melatonin) to address multifactorial PD-RBD pathology while minimizing adverse effects (94, 95).

Although our study provides a comprehensive analysis of metabolic profiling and identifies potential biomarkers in PD and PD-RBD, it had several limitations. Firstly, PD and PD-RBD were diagnosed based on clinical criteria. To address this, future studies should link pathophysiology markers, genetic technology, and neuroimaging to enhance diagnostic specificity. Secondly, the genetic background, dietary habits, and lifestyle factors of the patients and HCs may have influenced metabolite levels. Future research should calibrate these variables in larger cohorts to improve robustness and reproducibility. Finally, the present study focused on serum metabolites, and further investigation should integrate genomics, transcriptomics, and proteomics to provide additional insights into the molecular mechanisms underlying PD.

In summary, the present study identified valuable serum metabolic alterations that distinguish PD patients from HCs and PD-RBD patients from PD-nRBD patients, implicating dysregulated pathways (e.g., CCM and PPAR signaling) in PD pathogenesis. The identified metabolites (e.g., S-adenosylmethionine and 3-methyl-L-tyrosine) offer the potential for diagnosing and monitoring disease progression, while PPAR modulation may address RBD-specific pathology in PD. These findings enhance the understanding of neurodegenerative processes in PD and may facilitate the discovery of therapeutic targets.

## Data Availability

The original contributions presented in the study are included in the article/Supplementary material, further inquiries can be directed to the corresponding author.

## Footnotes

^1^ http://www.metaboanalyst.ca
